# Pesticide use, erythrocyte acetylcholinesterase level and self-reported acute intoxication symptoms among vegetable farmers in Nepal: a cross-sectional study

**DOI:** 10.1186/1476-069X-13-98

**Published:** 2014-11-20

**Authors:** Dinesh Neupane, Erik Jørs, Lars Brandt

**Affiliations:** Unit of Health Promotion, University of Southern Denmark, Esbjerg, Denmark; Nepal Development Society, Chitwan, Nepal; Clinic of Occupational Medicine, Odense University Hospital, Odense, Denmark

**Keywords:** Farmers, Carbamates, Erythrocyte acetylcholinesterase, Haemoglobin, Organophosphates, Pesticides, Nepal

## Abstract

**Background:**

As pesticide use is increasing and proper handling training is lacking, exposure to pesticides and intoxications are an important public health problems among farmers in developing countries. This study describes pesticide use among farmers and compares symptoms of possible acute intoxication and Erythrocyte Acetylcholinesterase(AChE) levels among vegetable farmers with a control group of blood donors in Nepal.

**Methods:**

A cross-sectional study was carried out among 90 pesticide-exposed farmers and a control group of 90 blood donors. Participants were randomly selected and data were gathered through questionnaires, observation and blood test. Chi-square test, logistic regression and Student’s t-test were used for data analysis to describe pesticide use and compare symptoms and AChE levels between the two groups. This study was approved by Nepal Health Research Council.

**Results:**

The majority of pesticides used were WHO class II, classified as moderately hazardous. The mean numbers of personal protective equipment used by farmers were 2.22 (95% CI: 1.89; 2.54). Out of five hygienic practices asked, farmers followed 3.63 (95% CI: 3.40; 3.86) hygienic practices on the average. Farmers reported more symptoms of possible pesticide intoxication in the past month than did controls, mean 5.47 (95% CI: 4.70; 6.25) versus 2.02 (95% CI: 1.63; 2.40) (p < 0.05). The mean haemoglobin-adjusted AChE(Q) was significantly lower among farmers compared to controls, 28.92 (95% CI: 28.28; 29.56) U/g versus 30.05 (95% CI: 29.51; 30.60) U/g, (p = 0.01). The risk of a farmer having lower Q level was about 3 times (OR = 2.95; 95% CI: 1.16; 7.51) greater than controls.

**Conclusion:**

Nepalese farmers exposed to pesticides have significantly more symptoms of possible pesticide intoxication than a control group of healthy individuals. A lower mean haemoglobin- adjusted AChE level was seen among farmers compared to the controls. The use of highly toxic pesticides, inadequate use of personal protective equipment and poor hygienic practices might explain the reason for symptoms of pesticide intoxication and a lower AChE level among farmers. Education and information of farmers should be undertaken to remediate these problems.

## Background

Nepal is predominantly an agrarian country. The agricultural sector employs over 66% of the working population and contributes to 39% of the gross domestic product [1]. In the last five decades pesticide use has increased sharply in the pursuit of increasing agricultural production. A third of agriculture production is destroyed in pre- and post-harvest operations due to pest attacks in the field and storage [2]. Thus farmers use pesticides disproportionately to avoid pest attacks. The pesticide use at national level for the year 2008 was 151.2 g active ingredient per hectare of arable land [3]. Pesticides are not as extensively used in Nepal in terms of active ingredients used per hectare of cropland compared to many other countries [4]. However, pesticide use in Nepal in terms of location, intensity, target crops, types of chemicals and increasing consumption suggests a risk of significant exposures and intoxications among farmers [5, 6].

The use of highly toxic pesticides is a major occupational risk among farmers in low income countries including Nepal [7]. Despite government’s and donors’ continuous efforts to promote Integrated Pest Management (IPM) [8] reliance on chemical pesticides has been growing in Nepal. Older, non-patented, more toxic, environmentally persistent and inexpensive chemicals are used extensively [9]. The safety measures recommended by Food and Agriculture Organisation of United Nation (FAO) are not followed in Nepal as in other low-income countries [10]. Few studies have been undertaken to explore different aspects of pesticides issues in Nepal [11, 12]. One study reported lower Acetylcholinesterase(AChE), levels during the ‘high pesticide use season’ as compared to the ‘low pesticide use season’ [11], but non-exposed controls were not included. Studies from other parts of the world were mostly based on self-reported symptoms of acute intoxication, often without laboratory tests or non-exposed control groups [13–26].

The aim of this study is to describe the types of pesticides used, the use of Personal Protective Equipment (PPE), and hygienic practices among farmers. The study also compares self-reported symptoms in the past month and AChE levels between farmers and controls.

## Methods

### Study design and setting

A comparative cross-sectional survey was conducted by collecting data from vegetable farmers and a control group of blood donors of Chitwan district of Nepal from April to June 2012. Chitwan covers an area of 2,205 square kilometers and had a population of 579,984 in 2011 [27]. The district was chosen because it is one of the main commercial and intensive vegetable cultivation areas with a high volume of pesticide usage. Agriculture is the primary source of income for the population of the district [27].

### Sample size and selection of participants

Most of the commercial vegetable farmers in Chitwan are associated to Fruit and Vegetable Farmers Cooperatives. A list of all Vegetable Farmers’ Cooperatives was obtained from the District Agriculture Development Office. Membership lists were obtained from the respective cooperatives in order to find participants. The households to visit were selected in consultation with Farmers Cooperatives based on population proportion to size. Only male farmers who owned at least 10 katthas (0.168 hectares) of land, had used pesticides within a year, were engaged in vegetables production, did not have any other profession and did not report any known conditions that could influence on AChE levels (e.g. paroxysmal nocturnal haemoglobinuria, macrocytic anemia, microcytic anemia or use of pyridostigmine) were eligible to be participant. Controls were chosen from blood donors who were occupationally non-exposed to pesticides and they were matched for sex, age group and district.

The estimated study sample size was calculated based on AChE values obtained by J Gomes and et al. among desert farm workers in the United Arab Emirates [19]. For a desired 95% confidence interval, considering alpha = 0.05 and design effect = 1.5, the sample size of 90 in each group was calculated to yield a power over 80%.

### Data collection

The investigator and a laboratory assistant visited each farmer to retrieve data by means of a questionnaire asked through face to face interview, observational checklist, height and weight measurement and blood test carried out on the same day. Information on demography, smoking habit, alcohol intake, type of pesticides used, use of PPE, hygienic practices and self-perceived symptoms were obtained from a questionnaire interview and an observational checklist which were developed by modifying the tools used to conduct a similar study in Bolivia [[18]]. For PPE, we asked availability and use of long-sleeve shirt, cap, mask, gloves, glasses, boot and gown and for hygienic practices we asked whether farmers often do practice hand washing with soap and water after spraying pesticides, changing clothes after spraying pesticides, hand washing with soap and water before eating during/after spraying pesticides, bathing whole body after spraying pesticide, smoking during a spraying session after handwashing with soap and water and immediate hand washing with soap and water after mixing pesticides. Regarding self-reported symptoms, we asked “Did you suffer from any of the following symptoms in the last month?” We also asked same question for them whether they experienced such symptoms immediately after spraying pesticides or not. We used WHO clinical symptoms of acute organophosphate and carbamates poisoning along with the previous Bolivian study to derive a list of symptoms [18, 28]. Some of the symptoms were difficult for farmer’s to report and therefore such clinical symptoms have been translated or combined into more understandable terms. The symptoms included in the questionnaire were: nausea, blurred vision, dizziness, skin allergy, excessive salivation, muscle cramp, headache, trembling hands, breathing difficulties, extreme tiredness, vomiting, abdominal pain, loss of appetite, lack of coordination, excessive sweating, difficulty in speaking and dry mouth.

The data for the control group were collected at the Regional Blood Transfusion Service Center, Bharatpur, Chitwan. They gave consent to participate in the study prior to screening for blood donation. The same questionnaire and measurements were used for controls. Literacy status was categorized in three categories (read easily, read with difficulty and cannot read) based on respondent capacity to read a sentence given during the interview.

Organophosphate or carbamate pesticides inhibit the blood enzymes AChE [29]. So, AChE measurement was taken for both farmers and control. Information on the level of AChE, Haemoglobin adjusted Erythrocyte Acetylcholinesterase Activity (Q) and haemoglobin was obtained from the blood test with the Test-mate ChE Cholinesterase Test System (Model 400) developed by EQM Research Inc [30]. In short, fingers were wiped with alcohol and then air-dried for about 30 seconds. Ten ml capillary blood was collected using a finger prick sterile lancing device and placed into the assay tube. AChE erythrocyte cholinesterase reagent was then dissolved in distilled water and inserted into the analyzer following the guidelines. The analyzer provided the reading for haemoglobin, AChE and Q, which were noted down in the same questionnaire. All laboratory tests were conducted in the field below 30°C.

### Data analysis

Data were entered in a database developed in Microsoft Access and imported to STATA 11 for analysis. For qualitative variables, chi-square test was used to see the difference between farmers and controls. Fisher’s exact test was applied for some variables when expected frequency in each cell was less than 5. Data were analyzed as two independent samples from normal distributions based on the Student's T-test. Estimates are given with 95% confidence intervals. Q was dichotomized into high and low categories. The cut-off point was one SD below the mean Q, i.e. at the 27.4. While selecting control and farmer we matched for five years age group (15-19, 20-24 and so on). For example, 35 years of farmer might have 39 years of control. That is why; we found statistically significant difference in mean ages between control and farmers. So, age was adjusted in further analysis. Regression analyses were performed adjusting for body mass index, age and literacy.

### Ethical consideration

Ethical approval for the study was obtained from Nepal Health Research Council. Written informed consent was obtained from all participating farmers and controls. Data were treated in a confidential manner with access only to the investigators and the laboratory assistant.

## Results

### Characteristics of respondents

The characteristics of farmers and controls are presented in Table 1. The average land area used for farming was 1.13 hectares and the average number of years involved in commercial vegetable farming was about 10 years. About 83% of the farmers had sprayed pesticides on an average of 6 hours during the week prior to the data collection date.

**Table 1:** **Characteristics of respondents**

Variable	Farmers (n = 90)	Control (n = 90)
Mean Age (years)	41.83	38.36
Respondent can easily read and write	50%	88%
Married respondents	91%	86%
Mean body mass index (kg/m^2^)	21.41	25.18
Smoking habit	23%	34%
Alcohol drinking habit	65%	33%
Mean land used by farmers (hectare)*	1.13(0.87; 1.39)	
Involvement in vegetable farming (years)	10.17(8.66; 11.69)	
Pesticide use years	10.30(8.86; 11.73)	

Numbers in parenthesis are for 95% CI, * = 86 because 4 respondents were professional sprayer.

### Type of pesticides

Organophosphate and carbamates were the most commonly used pesticides accounting for 66% of total pesticide use. Seven percent of pesticides belonged to organochlorine group. The remaining 27% were pyrethroid, macrocyclic lactone, anthranilicdiamide and unclassified. Seventy-one percent of farmers (n = 64) had pesticides in stock, and among them, each farmer had an average of 2 pesticides (range: 1 to 28). The top ten most common pesticides used by farmers are presented in Table 2. Out of the total pesticides in stock, 21% had expired while expiration date was not mentioned in 19%. Thus 41% of the stored pesticides were obsolete. When classified according to WHO criteria, 50% of pesticides were classified as moderately hazardous (II); 15% as highly hazardous (Ib) and 13% as slightly hazardous (III) categories. Only 6% of pesticides were unlikely to represent any acute hazard (U) in normal use.

**Table 2:** **Ten most common pesticides used in the study area (N = 64)**

Common name	Frequency	Percent	Chemical classification	Type of pesticide	WHO category
Chlorpyriphos 50% and Cypermethrin 5% EC	49	76	Organophosphate + pyrethroid	Insecticide	II
Imidaclorprid 17.8%	33	51	Organophosphate	Insecticide	II
Flubendiamide	17	29	Anthranilicdiamide	Insecticide	NL
Dichlorvos 76% EC	16	25	Organophosphate	Insecticide	Ib
Propargite 70% EC	13	20	Unclassified	Insecticide	III
Endosulfan	13	20	Organochlorine	Insecticide	II
Methomyl 40	12	18	Carbamate	Insecticide	IB
Mancozeb	11	17	Dithiocarbamate	Fungicide	U
Emamectin Benzoate	10	17	Macrocyclic Lactone	Insecticide	III
Cypermethrin 10%	10	17	Pyrethroid	Insecticide	II

Note: Only pesticides found during the home visit were recorded.

Nearly half of the interviewed farmers (44%) had stored pesticides easily accessible by children, while 25% stored pesticide in a closed containers. Fifteen percent of the respondents stored pesticides in their farmhouse. About 7% respondents stored pesticides in the kitchen and the same percentage stored pesticides in the sleeping room. About 36% of the farmers stored pesticides in the ceiling of their animal shed in a polythene bag. It was observed that all pesticides were stored in original boxes/containers.

### Use of personal protective equipment and hygienic practices

A total of 13% farmers did not use any PPE while spraying pesticides in the field. On an average, farmers used about 2 PPE. Cap was the most commonly used protective measure (64%) followed by long sleeved shirt (56 %). About 46% farmers used dust mask to protect against pesticide while spraying in the field and 33% farmers used long legged trousers or pants. The percentage of respondents who used glass, gowns, boots and gloves was less than 10%. Through observation, it was confirmed that 95% of farmers had long sleeve shirts, 63% of them possessed a mask, 31% had gloves and 21% had boots. The mean number of PPE used was 2.22 (95% CI: 1.89; 2.54). Fifty percent of those who could read easily used more than 2 PPE compared to 26% who did not, (p < 0.01). No significant differences was seen for different age groups as 73% of those whose age was less than the mean age (41.83 years) used more than 2 PPE compared to 66% of those whose age was higher than the mean age (p = 0.30).

Of the 5 hygienic practices, farmers on the average followed 3.63 (95% CI: 3.40; 3.86). Forty percent of the farmers (n = 36) washed their hands with soap and water immediately after mixing pesticides while 96% respondents washed their hands after spraying pesticides. Seventy-two percent (n = 65) of the interviewees replied that they washed their hands after spraying pesticides before eating. Those respondents who did not wash their hands before eating after spraying in the field were mostly putting chewing tobacco in their mouth. About 70% of the respondents washed their whole body after finishing spraying pesticides. Similarly, more than 84% of the farmers changed their clothes when they finished spraying in the field. Four percent respondents mentioned that they sprayed pesticide with the wind direction. Two-third of the farmers reported that their sprayer leaked. Among smokers, only 50% washed their hands before smoking. Sixty percent of those who could read easily used more than 2 hygienic practices as compared to 32% of those who could not read easily, (p < 0.01). Likewise, 37% of those younger than the mean age (41.8 years) practiced more than 2 hygienic practices compared to 48% of those above the mean age (41.8 years), (p = 0.13).

One in two respondents either sold empty pesticide bottles to Kawadi (ragpickers) or disposed them in a municipality container. None of the respondents reported that they reused empty pesticide containers/bottles/bags/boxes. However, 44% respondents said that they threw the containers in the field, around the houses or nearby rivers. Twelve percent of the farmer’s burnt the empty used pesticide bottles/bags/containers. We did not find any statistically significant association between ability to read and correct method of disposal.

### Possible acute intoxication symptoms

On the average, farmers reported 4.78 (95% CI: 4.05; 5.52) possible symptoms of acute intoxication in the previous month compared to the controls, who reported 1.58 (95% CI: 1.25; 1.92) (p < 0.05) (Table 3). Farmers reported about 7.28 (95% CI: 6.40; 8.16) symptoms immediately after handling pesticides throughout their lifetime. The most often reported symptoms among farmers in the previous month were blurred vision (50%) and extreme tiredness (47%). Those who experienced symptoms immediately after spraying when farming were more likely to have experienced symptoms in the past month (p < 0.01). Logistic regression analysis adjusted for age, body mass index and literacy showed odds ratio consistently higher among farmers as compared to controls [Table 4]. The highest and lowest odds ratios were observed for the variables ‘lack of coordination’ (OR = 6.27) and ‘loss of appetite’ (OR = 1.74) respectively (Table 4).

**Table 3:** **Reported symptoms by farmers (in the last month and immediately after pesticide use) and controls (in the last month)**

Symptoms	In the last month			Ever experienced any symptoms immediately after pesticide use
	Farmers (%)	Controls (% )	P-value	Farmers (%)
	(n = 90)	(n = 90)		(n = 90)
Nausea	25	8	<0.01	47
Blurred vision	50	16	<0.01	70
Dizziness	34	6	<0.01	56
Skin Allergy	25	17	0.159	50
Excessive Salivation	3	3	1.0 ^x^	5
Muscle cramps	40	16	<0.01^x^	51
Headache	40	8	<0.01	55
Trembling hands	24	8	<0.01	33
Difficulty in breathing	21	4	<0.01^x^	30
Extreme tiredness	47	20	<0.01	70
Vomiting	6	1	0.11^x^	20
Abdominal pain	23	5	<0.01	36
Loss of appetite	24	6	<0.01	35
Lack of coordination	22	5	<0.01	31
Excessive sweating	43	17	<0.01	61
Difficulty in speaking	8	1	0.03^x^	18
Dry mouth	35	7	<0.01	46

^x^ = Fisher's exact test.

**Table 4:** **Odds ratio for the self reported symptoms among farmers (n = 90) as compared to controls (n = 90) in the past month**

Symptoms	OR (95% CI)
Nausea	3.10(1.01-9.55)
Blurred vision	7.25(2.65-19.80)
Dizziness	3.54(1.18-10.56)
Skin Allergy	1.77(0.70-4.42)
Muscle cramps	3.36(1.33-8.51)
Headache	5.03(1.82-13.85)
Trembling hands	2.49(0.78-8.03)
Extreme tiredness	2.58(1.10-6.07)
Abdominal pain	1.86(0.53-6.45)
Loss of appetite	1.74(0.48-6.28)
Lack of coordination	6.27(1.64-23.99)
Excessive sweating	3.32(1.27-8.69)
Dry mouth	5.06(1.72-14.86)

Note: Adjusted for age, body mass index and literacy. Not included in analysis: salivation, breathing difficulties, vomiting and speak difficulties because of few values in each cells.

There was no statistically significant difference between farmers and controls in terms of health care seeking behavior. Out of 90 farmers, 87 (97%) felt sick in the previous month, and 45 (50%) of them visited health care facility whereas out of 90 control, 57 (63%) felt sick in the previous month, and 65% of them visited health care facility. Among 140 respondents who had reported that they felt sick in the last month prior, 83 were farmers and 57 were controls. Among them, about 50% of the farmers and 65% of the controls visited a health care facility. Sickness self-management during illness was higher among controls (29%) as compared to farmers (22%).

### Acetyl cholinesterase levels

AChE, heamoglobin and Q levels were significantly lower among farmers as compared to the controls. Logistic regression analysis adjusting for body mass index, age, literacy status and alcohol showed that a farmer’s risk of having a lower Q level is about 3 times (OR = 2.95; 95% CI: 1.16; 7.51) the risk of the controls. Though, participants who reported at least one acute intoxication symptom had -1.02(95% CI: -2.06; 0.028) unit less Q as compared to those who did not report any acute intoxication symptoms from linear regression analysis, there was no statistically significant association between participants reporting at least one acute intoxication symptoms and Q (Table 5).

**Table 5:** **Mean AChE level of farmers and control**

Variables	Mean (95% CI)		P-value
	Farmers	Control	
AChE (U/mL)	3.35(3.24; 3.45)	3.64(3.53; 3.75)	<0.01
Q (U/g)	28.92(28.28; 29.56)	30.05(29.51; 30.60)	<0.01
Haemoglobin (g/dL)	11.58(11.32; 11.84)	12.12(11.81; 12.44)	<0.01

## Discussion

This study found widespread use of moderately hazardous pesticides WHO class II and limited use of proper PPE and hygienic practices. An earlier study among Nepali farm workers showed low levels of pesticide handling practices when using pesticides even though the majority of farmers were knowledgeable about possible pesticide harm [11]. The infrequent use of PPE has been reported by studies conducted in other low-income countries as well [13, 18, 20-22 ]. Studies mentioned that the low use of PPE may be due to low education level, lack of training, low income, pesticide dealers not promoting/selling PPE, limited awareness and discomfort [13, 18, 20–22]. The use of dust masks, caps and long-sleeve shirts by the majority of farmers means that farmers are willing to wear them might signal their willingness to adopt other, more evidence-based measures. This could be taken as an opportunity to introduce similar but more effective equipment for prevention of pesticide exposure among farmers. With the exception of hand washing with soap after spraying limited hygienic practices were found among farmers similar to studies from Cambodia [21] and Ethiopia [26] but in contrast to studies from Bolivia [[18]] and Oman [22].

We found that farmers had more symptoms in the past month compared to controls. Some of this excess may be related to pesticide exposure, but could also be due to dehydration, exhaustion from work and other factors. Symptoms related to heavy work and lack of fluids (sweating, difficulty breathing, thirst) were also higher among farmers compared to control. Inadequate PPE use, poor hygienic practices and the use of highly toxic pesticides have probably increased pesticide exposure and related symptoms as other study found that the use of protective measures was associated with fewer intoxication symptoms after handling pesticides [[18]]. Other studies have consistently reported more symptoms among pesticide-using farmers as compared to controls, results that support our finding of pesticides probably being responsible for the excess symptoms among the farmers compared to controls [14, 18, 19]. We could not find statistically significant association between reported acute intoxication symptoms and decreased Q level. This could be because our exposure measurement was crude and symptoms were self-reported by participants. A study found that cholinesterase inhibition was associated with symptoms from the respiratory system, eyes and central nervous system among farmers [14]. Thus, the significantly lower AChE level among farmers compared to controls further suggests that the symptoms could partly be due to pesticide exposures. Though the majority of farmers mentioned that they had at least one possible symptom of acute intoxication during one month prior to our interview, 50% of them visited the health facility to seek care. However, there is a lack of specific training among health professionals regarding pesticide intoxication.

Our study supports the finding that farmers in developing countries do not store pesticides in safe places and that children may have easy access to them. Studies conducted in Nepal, Oman and Cambodia showed similar result [11, 21, 22]. Easy access by children means that children are at a higher risk of accidental intoxication. Pesticides stored in the sleeping room, kitchen, shed, store room and in the attics are easy accessible when needed - but also provides easy access when suicide is contemplated. Many studies have shown that easy access to pesticides is directly linked to suicides [31]. In addition evidence suggests that restricting access to lethal pesticides significantly reduces suicide rates [32]. Another major problem described in our study was the improper disposal of used pesticide containers, which might lead to environmental pollution and intoxications. This finding is similar to studies from other countries though some differences were observed concerning disposal methods [20, 22, 33]. A case-control study showed that exposure to used containers containing pesticides was associated with an increased birth defect risk [34].

### Limitations

Controls had no known pesticide exposure. However, they might have been exposed to pesticides due to living near farms where pesticide was used, while passing through sprayed or more likely from contaminated food. However, this would lead to bias toward the null.

The use of blood donors as controls is likely to introduce selection bias as blood donors generally healthier than the general population. Nepal blood transfusion services follow are as per WHO advocacy and recommendations, which is based on voluntary non-remunerated regular blood donation, which will minimized the any potential bias that the controls could have a lower socio-economic status [35]. The study tried to minimize these limitations by matching for sex and geographical region when recruiting controls and by adjusting for age in the multivariate analysis. Recall bias on the part of farmers cannot be ruled out as information was obtaining through interviews.

## Conclusions

Nepalese farmers exposed to pesticides have significantly more symptoms of possible pesticide intoxication than a control group of healthy individuals. A lower mean haemoglobin- adjusted AChE level was seen among farmers compared to the controls. The use of highly toxic pesticides, inadequate use of personal protective equipment and poor hygienic practices might explain the reason for symptoms of pesticide intoxication and a lower AChE level among farmers. In spite of many years of promoting IPM to Nepali farmers there is still an urgent need for educating farmers in improved pesticide handling techniques and IPM alternatives to protect the health of themselves and their families.

## Abbreviations

AChE: Erythrocyte Acetylcholinesterase
FAO: Food and Agriculture Organisation of United Nation
IPM: Integrated Pest Management
PPE: Personal protective equipment
Q: Haemoglobin adjusted Erythrocyte Acetylcholinesterase Activity
WHO: World Health Organisation.
